# Is *Helicobacter pylori* infection associated with non-alcoholic fatty liver disease in individuals undergoing bariatric surgery? Cross-sectional study

**DOI:** 10.1590/1516-3180.2022.0517.R1.14122022

**Published:** 2023-04-07

**Authors:** Erick Coelho Valadares, Martinho Antonio Gestic, Murillo Pimentel Utrini, Felipe David Mendonça Chaim, Elinton Adami Chaim, Everton Cazzo

**Affiliations:** I MD. Resident Physician, Department of Surgery, School of Medical Sciences, Universidade Estadual de Campinas (UNICAMP), Campinas, Brazil.; II MD, MSc. Assistant Physician, Department of Surgery, School of Medical Sciences, Universidade Estadual de Campinas (UNICAMP), Campinas, Brazil.; III MD. Assistant Physician, Department of Surgery, School of Medical Sciences, Universidade Estadual de Campinas (UNICAMP), Campinas, Brazil.; IV MD, PhD. Assistant Physician, Department of Surgery, School of Medical Sciences, Universidade Estadual de Campinas (UNICAMP), Campinas, Brazil.; V MD, PhD. Full Professor, Department of Surgery, School of Medical Sciences, Universidade Estadual de Campinas (UNICAMP), Campinas, Brazil.; VI MD, PhD. Associate Professor, Department of Surgery, School of Medical Sciences, Universidade Estadual Campinas (UNICAMP), Campinas, Brazil.

**Keywords:** Helicobacter pylori, Non-alcoholic fatty liver disease, Obesity, Metabolic syndrome, Bariatric surgery, Campylobacter pylori, NAFLD, RYGB

## Abstract

**BACKGROUND:**

A possible direct link between nonalcoholic fatty liver disease (NAFLD) and *Helicobacter pylori* (*H. pylori*) infection has recently emerged.

**OBJECTIVE:**

This study aimed to analyze associations between the presence of histologically demonstrated NAFLD aspects with *H. pylori* infection in individuals with obesity undergoing bariatric surgery.

**DESIGN AND SETTING:**

An observational analytical cross-sectional study was conducted based on data collected from the medical records of individuals undergoing bariatric surgery at a tertiary university hospital in 2019.

**METHODS:**

NAFLD was assessed through histological examination of wedge liver biopsies collected during the proceedings. *H. pylori* infection was analyzed through the association of the urease test and histological examination performed in biopsies routinely collected during preoperative esophagogastroduodenoscopy.

**RESULTS:**

Of the 88 participants, 85% were female, and the average age was 39.1 ± 8.4 years. *H. pylori* infection was present in 61.4% of the patients. The mean body mass index was 36.6 ± 3.4 kg/m^2^. The most prevalent histopathological aspects of NAFLD were macrovesicular steatosis (92%), hepatocellular ballooning (92%), lobular inflammation (93.2%), portal inflammation (96.6%), and fibrosis (93.2%). No histopathological aspect of NAFLD was found to be significantly associated with *H. pylori* infection.

**CONCLUSION:**

In this study population, H. pylori infection was not significantly associated with the histopathological aspects of NAFLD in individuals with obesity undergoing bariatric surgery.

## INTRODUCTION

The association between non-alcoholic fatty liver disease (NAFLD) and extrahepatic conditions has been increasingly reported in recent years, and its correlation has been described with conditions such as obesity, metabolic syndrome, diabetes, sarcopenia, heart disease, and chronic kidney disease. Furthermore, a possible direct link between NAFLD and *Helicobacter pylori* (*H. pylori*)infection has emerged. The mechanisms of this association, however, remain unclear and seem to be associated with low-grade inflammation underlying chronic infection by this bacterium and its connection with insulin resistance and imbalances in lipid metabolism.^1,2^Meta-analyses carried out by Mantovani et al.^3^ and Wei et al.^4^ suggested a significantly increased risk of NAFLD among *H. pylori* carriers. Nonetheless, these reviews included studies that assessed NAFLD using heterogeneous diagnostic methods, primarily imaging techniques.

This study aimed to analyze the association between the presence of histologically demonstrated NAFLD and *H. pylori* infection in individuals with obesity undergoing bariatric surgery (BS).

## METHODS

### Study design

An observational, analytical, cross-sectional study was conducted based on data collected from medical records of individuals undergoing BS at a tertiary university hospital in 2019. The study protocol was evaluated and approved by the local institutional review board under opinion 4.677.470 (CAAE: 45210321.8.0000.5404; date: April 28, 2021). All the participants provided informed consent.

### Study population

This study included individuals aged 18 to 70 years, of any sex, who underwent Roux-en-Y gastric bypass(RYGB) according to the National Institutes of Health criteria. Exclusion criteria were history of other liver diseases, cholestatic diseases and viral hepatitis, belonging to vulnerable groups (underaged or with severe mental or intellectual impairment), recent or current use of alcohol, illicit drugs or hepatotoxic medications, and incomplete medical records.

Of the101 individuals who underwent RYGB, 88 were selected for the study; 13 individuals were excluded for viral hepatitis (n = 2), hepatotoxic medications (n = 3), previous cholestasis (n = 1), and incomplete medical records (n = 7).

### Demographic, anthropometric, clinical, and biochemical data

Data regarding age, sex, body mass index (BMI), and presence of hypertension and type 2 diabetes were collected. The following laboratory parameters were analyzed in this study: fasting glucose (mg/dL), aspartate aminotransferase (AST, IU/L), and alanine aminotransferase (ALT, IU/L).

### NAFLD assessment

NAFLD was assessed through histological examination of wedge liver biopsies collected during the procedure. The main NAFLD features were classified into following categories:1) macrovesicular steatosis; 2) microvesicular steatosis; 3) hepatocellular ballooning; 4) lobular inflammation; 5) portal inflammation); and6) fibrosis. These aspects were classified as absent or present according to the classification system proposed by Brunt et al.^5^ (Reviewer #1; Comment #2) Biopsies were systematically performed in all bariatric operations at this facility as part of routine care. The histopathological examination was performed by the same pathology team.

### Helicobacter pylori infection assessment

*H. pylori* infection was analyzed by means of a urease test and histological examination with Giemsa stain was performed on biopsies that were routinely collected during preoperative esophagogastroduodenoscopy. *H. pylori* infection was classified as present or absent.

*H. pylori* infection status was correlated with the presence and severity of NAFLD aspects above cited.

### Statistical analysis

To compare proportions, the chi-square test or Fisher’s exact test was used, when necessary. The Mann–Whitney test was used to compare continuous variables. The significance level was set at 5% (P < 0.05). Analyses were performed using the SAS System for Windows (Statistical Analysis System, version 9.2; SAS Institute Inc., 2002-2008, Cary, North Carolina, United States).

## RESULTS

### Demographic, anthropometric, and clinical data

Ofthe88 participants, 85% were female, and the average age was 39.1 ± 8.4 years. *H. pylori* infection was present in 61.4% of the patients. The mean BMI was 36.6 ± 3.4 kg/m^2^. Hypertension was present in 40.9% of patients, and 22.7% presented with diabetes. There were no significant differences between these variables in individuals with or without *H. pylori* infection.

### Biochemical variables

The mean AST levels were 22.4 ± 8 IU/L and ALT levels were 27.4 ± 14.7 IU/L. The average fasting glucose was 88.9 ± 20.1 mg/dL. There were no significant differences in biochemical variables between individuals with or without *H. pylori* infection.

### Histopathological features

The most prevalent histopathological aspects of NAFLD were macrovesicular steatosis (92%), hepatocellular ballooning (92%), lobular inflammation (93.2%), portal inflammation (96.6%), and fibrosis (93.2%). No histopathological aspect of NAFLD was significantly associated with *H. pylori* infection.

Complete comparisons between individuals with and without *H. pylori* infection are shown in Table 1.

**Table 1.** Comparison of demographic, anthropometric, clinical, biochemical, and NAFLD-related histopathological aspects between individuals with or without *H. pylori* infection

	*H. pylori* infection	No *H. pylori* infection	P value
N	54 (61.4%)	34 (38.6%)	NA
Age (years)	39.1 ± 11	39.4 ± 8.1	0.9
Gender	Female: 48 (88.9%) Male: 6 (11.1%)	Female: 27 (79.4%) Male: 7 (20.6%)	0.2
BMI (kg/m^2^)	37.8 ± 6.4	36.5 ± 0.6	0.8
Hypertension	20 (44.4%)	16 (47.1%)	0.4
Type 2 diabetes	9 (16.7%)	11 (32.4%)	0.1
AST (IU/L)	22.6 ± 8.4	22.2 ± 7.4	0.8
ALT (IU/L)	28.1 ± 16.5	26.4 ± 11.8	0.6
Fasting glucose (mg/dL)	90.1 ± 19.1	87.1 ± 21.6	0.5
Macrovesicular steatosis	49 (90.7%)	32 (94.1%)	0.6
Microvesicular steatosis	19 (35.2%)	11 (32.4%)	0.8
Hepatocellular ballooning	50 (92.6%)	31 (91.2%)	0.8
Lobular inflammation	48 (88.9%)	34 (100%)	0.1
Portal inflammation	52 (96.3%)	33 (97.1%)	0.8
Fibrosis	50 (92.6%)	32 (94.1%)	0.8

*H. pylori = Helicobacter pylori*; NAFLD = non-alcoholic fatty liver disease; N = number of individuals; NA = not applicable; BMI = body mass index; AST = aspartate aminotransferase; ALT = alanine aminotransferase.

## DISCUSSION

In the current study, the occurrence of both *H. pylori* infection and NAFLD was considerably high in individuals undergoing BS. These findings are comparable to those of the previous studies. There were no significant associations between *H. pylori* infection and anthropometric, clinical, biochemical, or histopathological aspects in the current population sample. The study population mainly comprised women (85%) aged between 30 and 50 years, which is common in BS. Fuchs et al.^6^ analyzed the sex gap in BS in the United States through a multicenter database comprising 190,705 individuals who underwent BS between 1998 and 2010 and found a similar 4:1 proportion, despite an almost 1:1 proportion of obesity between males and females. The authors attributed this gap to several factors, highlighting a slightly greater eligibility for BS among women alongside some degree of sociocultural pressure regarding weight loss within this gender group and a lower willingness to seek medical care among men. Furthermore, this study pointed out that the gender gap is more prominent in lower-income populations.

*H. pylori* infection has been suggested to play a role in the pathogenesis of insulin resistance by several mechanisms, mostly through increased levels of pro-inflammatory cytokines, eicosanoids, acute phase proteins, reactive oxygen species production, and changes in serum cytokines.^7, 8, 9, 10^ Recent studies have shown that *H. pylori* also plays a potential role in systemic chronic inflammation by increasing intestinal permeability.^11, 12, 13^ These mechanisms are also reported to be directly related to the development of NAFLD.^1,14, 15, 16^

Some high-quality studies have reported this association. A large cross-sectional study carried out by Jiang et al.^17^ with 4,081 individuals identified a positive correlation between NAFLD diagnosed by ultrasound and *H. pylori* infection evaluated through urease breath test, mostly among females and individuals with dyslipidemia. Ina large prospective cohort study that included 17,028 participants initially free of NAFLD, Kim et al.^18^ demonstrated that *H. pylori* infection was independently associated with the incidence of *“de novo”* NAFLD. These findings were reinforced by the meta-analyses by Mantovani et al.^3^ and Wei et al.^4^

Nevertheless, the currently available literature is far from consensus on the existence of this positive association between *H. pylori* and NAFLD, with other methodologically appropriate studies demonstrating opposite findings. In large studies carried out in Japan by Okushin et al.^19^ and in China by Fan et al.^20^ with 13,737 and 21,456 participants, respectively, *H. pylori* was not an isolated risk factor for NAFLD. A study carried out by Baeg et al.^21^ that identified *H. pylori* infection as a variable significantly correlated with metabolic risk factors, including high BMI, blood pressure, triglycerides, and low HDL, failed to demonstrate an independent association between *H. pylori* and NAFLD. Furthermore, a prospective study by Jamali et al.^22^ showed that *H. pylori* eradication per se does not affect liver fat content and lipid profile in dyspeptic patients with NAFLD.

Considering that most population studies included samples of individuals with heterogeneous BMI status, this also raises the question of whether this putative association could be more or less likely to be identified among individuals with or without obesity. Lecube et al.^23^ analyzed a population of 416 individuals with both obesity and NASH and concluded that in patients with morbid obesity, *H. pylori* infection does not seem to be associated with abnormal carbohydrate metabolism and suggested that the low-grade inflammation that accompanies obesity seemingly mitigated the diabetogenic effect of *H. pylori*. In contrast, Doulberis et al.,^24^ investigating the metabolic burden of *H. pylori* infection in 64 morbidly obese individuals, observed that *H. pylori* infection was independently associated with insulin resistance, NASH, and liver fibrosis. Thus, even in this setting, the answer seems far from a consensus.

The current study has some limitations that should be considered. Its cross-sectional design did not provide insights into causal or consequential links. Since it included individuals undergoing BS, there was a tendency towards a very homogeneous population in relation to BMI status. Furthermore, this population has a high prevalence of NAFLD. Prospective studies enrolling individuals without obesity could clarify this issue. Considering that most previous studies were carried out in Asia and the current study was performed in a population of highly multi-ethnic heritage in South America, ethnicity may also play a role in the conflicting results. On the other hand, this study has the clear strength of analyzing NAFLD through the best possible method, that is, histopathological examination, which provided a detailed evaluation that imaging methods are unable to equally provide and, thus, adds more accurate information to the currently available evidence on this relevant topic.

## CONCLUSIONS

In the studied population, *H. pylori* infection was not significantly associated with either histopathological or biochemical variables of NAFLD in obese individuals undergoing BS.
